# Mathematical modeling reveals spontaneous emergence of self-replication in chemical reaction systems

**DOI:** 10.1074/jbc.RA118.003795

**Published:** 2018-10-03

**Authors:** Yu Liu, David J. T. Sumpter

**Affiliations:** From the Department of Mathematics, Uppsala University, 75105 Uppsala, Sweden

**Keywords:** chemical biology, computer modeling, catalysis, probiotic, bioenergetics, artificial chemistry, biological complexity, collectively catalytic, origin of life, self-replication

## Abstract

Explaining the origin of life requires us to elucidate how self-replication arises. To be specific, how can a self-replicating entity develop spontaneously from a chemical reaction system in which no reaction is self-replicating? Previously proposed mathematical models either supply an explicit framework for a minimal living system or consider only catalyzed reactions, and thus fail to provide a comprehensive theory. Here, we set up a general mathematical model for chemical reaction systems that properly accounts for energetics, kinetics, and the conservation law. We found that 1) some systems are collectively catalytic, a mode whereby reactants are transformed into end products with the assistance of intermediates (as in the citric acid cycle), whereas some others are self-replicating, that is, different parts replicate each other and the system self-replicates as a whole (as in the formose reaction, in which sugar is replicated from formaldehyde); 2) side reactions do not always inhibit such systems; 3) randomly chosen chemical universes (namely random artificial chemistries) often contain one or more such systems; 4) it is possible to construct a self-replicating system in which the entropy of some parts spontaneously decreases, in a manner similar to that discussed by Schrödinger; and 5) complex self-replicating molecules can emerge spontaneously and relatively easily from simple chemical reaction systems through a sequence of transitions. Together, these results start to explain the origins of prebiotic evolution.

## Introduction

Self-replication is one of the central properties of life (1), and to explain life's origins, we need to explain how self-replication arose. It is widely accepted that before DNA, life replicated through RNA molecules (2). However, it remains unclear how the building blocks of RNA, such as nucleotides, became available on the primitive Earth, and even if these building blocks were abundant, it is unclear how they were assembled into the first RNA (3, 4). It is plausible that self-replication did not originate from a single complex independent self-replicating molecule. In the early stage of evolution, the “precursor life” could be very different from what we see today (3, 5). For example, in Wächtershäuser's iron-sulfur world hypothesis, the precursor life does not have nucleic acids but consists of a self-replicating (or “autocatalytic,” in his words) metabolic network (6). Another proposal, by Szathmáry (7–9), is that life evolved from “holistic limited hereditary replicators” such as the formose reaction—in which sugar is replicated from formaldehyde—to “modular unlimited hereditary replicators” such as RNA and today's DNA.

There are lots of biological examples of self-replicating systems that do not rely on a single complex template molecule (as is the case for DNA and RNA molecules). These include the malic acid cycle (a metabolic path of some bacteria and plants for synthesis of malates), the Calvin cycle in photosynthesis (10), the reductive citric acid cycle for a certain group of chemoautotrophs (11, 12), the artificially designed myokinase–pyruvate kinase cycling system (13), the metabolic pathways of ATP (as well as some other coenzymes such as NAD^+^ and CoA) found in many different living organisms (14), and the whole metabolic reaction network of *Escherichia coli* (15). Self-replication has also been identified in nonliving systems, such as the formose reaction (10, 16), and experiments in laboratories (17–19), including self-replication of nucleotide-based oligomers (20).

Many models have been put forward to explain the origin of self-replication in terms of a system of coupled chemical reactions (21). Some of these can be categorized as artificial chemistry models, as reviewed by Dittrich *et al.* (22) and by Banzhaf and Yamamoto (23). For example, the chemoton model describes a system composed of three coupled quasi-self-replicating subsystems (metabolism, membrane, and template), which as a whole is able to self-replicate (10). The chemoton can be considered as a model of a minimal living system, but cannot explain how this system spontaneously develops from a soup of simple molecules. The metabolically coupled replicator system is another type of model in this direction (24, 25). But it mainly focuses on the information heredity and ecological stability, that is, how to maintain coexistence of different replicators and constrain parasitism, rather than investigating why self-replication can occur in the first place. The most influential model in this direction is the reflexively autocatalytic and food-generated (RAF)^2^ theory (26–28), extended from Kauffman's autocatalytic sets theory (29). A set of chemical reactions is RAF if 1) every reaction in this set is catalyzed by at least one molecule involved in this set and 2) every molecule involved in this set can be produced from a small food molecule set. RAF sets are shown to be able to readily emerge from a set of chemical reactions and always consist of smaller RAF sets, demonstrating the capability to evolve (30, 31). Other similar models also contributed to this theory (32–36), including the chemical organization theory (37) and the graded autocatalysis replication domain model (38). The former is closely related to RAF theory (see Hordijk *et al.* (39) for a detailed comparison), whereas the latter suffers from lacking evolvability (see Vasas *et al.* (40, 41) for detailed critical analyses). In addition, many of the biological observations mentioned in the previous paragraph can be put into the framework of RAF theory (15, 17–20).

Although RAF theory is influential, it has limitations as an explanation of self-replication. First, the theory stipulates that every reaction in the set is catalyzed. Even though catalyzed reactions are very common in living systems, not every reaction involves a catalyst (*e.g.* condensation reactions (8, 42)). In the early stages of biological evolution, probably no reaction required sophisticated biotic catalysts (*e.g.* enzymes) (11, 15), so there is a strong motivation not to include these enzymes (20, 43, 44) or even catalysts as given in a model of the origins of self-replication. Uncatalyzed reactions have significant effects on the dynamics of the whole system (*e.g.* “innovating” new species of molecules and then triggering other RAF sets) (45, 46). The second concern with RAF theory is that it is a purely graph-theoretic approach. As a result, there is no constraint on how chemical reactions are constructed and coupled (although it gives the theory much freedom, the construction of reaction systems is too arbitrary to investigate the model systematically). As another result, extra assumptions need to be made about chemical kinetics to investigate the dynamics of how populations of molecules change over time (47). Here, we see another reason to relax the assumption of studying only catalyzed reactions: catalyzed reactions are never elementary reactions, so the kinetics cannot be simply calculated from the reaction stoichiometry (48).

Any theoretical approach to the origin of self-replication should explicitly include energetics (15, 49), an aspect that is missing from all of these models and theories above. There are several reasons for this. First, energetics (*e.g.* Gibbs energy) determines whether a chemical reaction is spontaneous, so to investigate the spontaneity of the emergence of life, it has to be considered. Second, to concretely discuss the issue—famously put forward by Schrödinger—that life maintains its order by feeding on “negative entropy” (50), energetics has to be explicitly taken into account, as entropy is negatively related to the thermodynamic free energy (which is Gibbs energy in the scenario of constant pressure and temperature (51)). It should be noted that the relationship between life and entropy is investigated in different ways and contexts (52–54). For example, Branscomb and Russell (53) explained the specific mechanisms to increase thermodynamic free energy in two real-world biochemical scenarios: the hypothesized alkaline hydrothermal vent (55) on the prebiotic Earth and the system where the ferredoxin I protein translocates protons. In the context of statistical physics, a lower limit was derived for the amount of heat generated in a nonequilibrium system where a process of self-replication occurs (54).

In this paper, we set up an artificial chemistry model, in the form of a general mathematical framework for chemical reaction systems, that properly accounts for energetics, kinetics, and the conservation law. Catalysts are not explicitly included in the model, but we later find that catalysis can emerge, along with self-replication and potentially complex molecules, in our system.

In what follows, we give an overview of our model. The specifications are given under “Theory” at the end of this paper. We would encourage the reader, on first reading, to watch a short movie included in the supporting information that illustrates how our model works, and see “Theory” directly afterward.

We model a well-mixed soup of molecules, each of which is defined by its integer mass, *i*. A molecule's type is thus denoted *ī*. Only synthesis reactions and decomposition reactions are possible. Only reactions that conserve mass can occur (*i.e.* the total mass on the reactant side adds up to that on the product side). Each type of molecule *ī* has its own standard Gibbs energy of formation *G*_*i*_^°^, that decides whether a reaction is spontaneous. Moreover, a reaction has to overcome an energy barrier (namely Gibbs energy of activation) to occur. It is called *low-barrier* if its energy barrier is low and its reaction rate is thus high or *high-barrier* otherwise. To define a specific chemical reaction system, we show a list of low-barrier (and spontaneous) reactions only (*e.g.* Scheme 1).

$$\begin{array}{c} \{\begin{array}{ccccccc} & & \bar{5} & \to & \bar{1} & + & \bar{4}\\ & & \bar{6} & \to & \bar{1} & + & \bar{5}\\ \bar{2} & + & \bar{4} & \to & \bar{6} & & \end{array}\\ \text{SCHEME}\;1 \end{array}$$

Many other reaction systems can also be constructed. Some of them are physically possible—meaning that there are proper *G*_*i*_^°^ values for all molecules involved so that all reactions listed are spontaneous, whereas some others are not. We are only interested in physically possible systems.

After that, we assume that there is an unlimited reservoir of resource molecules (*e.g.* molecule 2̄ for system shown in Scheme 1). Then we use the standard Gillespie algorithm to simulate the dynamics, namely *N_i_*(*t*), the time series of molecule *ī*'s population. In addition, we construct ordinary differential equations (ODEs) to describe the mean-field dynamics.

## Results

### Collectively catalytic system

**Figure 1.**
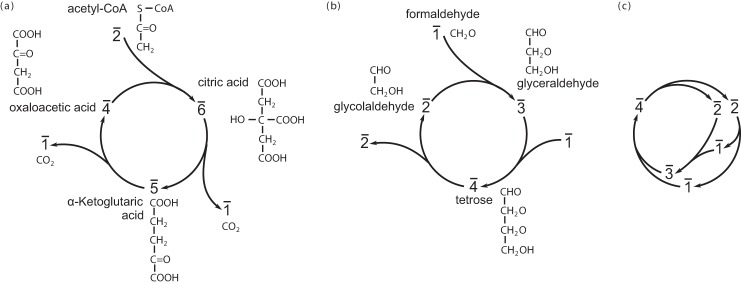
**Sketches of three chemical reaction systems specified within our model.** Details are described under “Theory.” *a*, a simplified model of the citric acid cycle. *b*, a simplified model of the formose reaction. In both simplified models, we only consider carbon-changing reactions and do not consider isomers of some molecules. Note that the integer representation of mass can be thought of as a rough ordering of the molecule's mass or relative complexity. Consequently, the addition operation representation for chemical reactions guarantees the mass conservation. *c*, a physically impossible chemical reaction system (Scheme 7).

We first investigate the system shown in Scheme 1, which is actually a model of the citric acid cycle in cellular respiration (10). Specifically, as shown in Fig. 1*a*, molecule 1̄ represents carbon dioxide, 2̄ represents acetyl-CoA, 4̄ represents oxaloacetic acid, 5̄ represents α-ketoglutaric acid, and 6̄ represents citric acid, respectively.

Given an unlimited reservoir of resource molecules 2̄, the simulation shows that *N*_1_(*t*) increases linearly. Also, molecules 4̄, 5̄, and 6̄ are involved in a cycle of reactions, but the total number is constant. Section S4 shows the dynamics and ODE solutions in more detail. We also observe that each reaction in Scheme 1 occurs approximately the same number of times. We can add up these reactions, cancel out the molecules appearing on both sides of the reaction (in this case, molecules 4̄, 5̄, and 6̄), and then obtain the overall reaction of the system 2̄ → 1̄ + 1̄. By comparison, the reaction 2̄ → 1̄ + 1̄ itself is high-barrier, and its reaction rate is thus extremely low, but through the whole system, the actual rate of the overall reaction is several billion times larger (section S4). We call the Scheme 1 system the *collectively catalytic system*, because the overall reaction 2̄ → 1̄ + 1̄ is catalyzed by molecules 4̄, 5̄, and 6̄. Note that this outcome is consistent with the biological observation that the citric acid cycle consumes acetyl-CoA (molecule 2̄) and produces carbon dioxide (molecule 1̄) as a waste product.

The linear growth of *N*_1_(*t*) occurs because 1) 1̄ is an end product that cannot be used by these low-barrier reactions; 2) the number of resource molecules 2̄ is constant; and 3) no additional molecules 4̄, 5̄, and 6̄ can be produced through these low-barrier reactions (by noting that the number of times molecules 4̄, 5̄, and 6̄ appear on the right-hand side of Scheme 1 is the same as that on the left-hand side).

We can use these observations to give a rigorous set of criteria for collectively catalytic systems. We start by defining an intermediate molecule to be any molecule that appears on both the reactant side and the product side. In general, the following stoichiometric criteria are sufficient (but not necessary) to show that a physically possible chemical reaction system, given supplies of resource molecules, is collectively catalytic: 1) for every low-barrier reaction, at least one type of its reactants comes from the products of other low-barrier reactions (called the *criterion for self-driven*), and 2) by adding up all of the low-barrier reactions, for every type of intermediate molecule, the number of times it appears on the reactant side and that on the product side are the same (called the *criterion for balanced-cancelling*).

The citric acid cycle (Scheme 1) thus satisfies all of these criteria, and there are other systems satisfying these criteria too (in fact, any single catalytic reaction can be written as a collectively catalytic system: see section S5 for details). The criteria above give us a way of discerning whether or not a system is collectively catalytic based on stoichiometry alone, without the need to investigate its dynamics.

### Self-replicating system

Now we investigate the formose reaction, which involves the formation of sugars from formaldehyde (10).

$$\begin{array}{c} \{\begin{array}{ccccccc} \bar{1} & + & \bar{2} & \to & \bar{3} & & \\ \bar{1} & + & \bar{3} & \to & \bar{4} & & \\ & & \bar{4} & \to & \bar{2} & + & \bar{2} \end{array}\\ \text{SCHEME}\;2 \end{array}$$

Specifically, as shown in Fig. 1*b*, molecule 1̄ represents formaldehyde, 2̄ is glycolaldehyde, 3̄ is glyceraldehyde, and 4̄ is tetrose, respectively.

**Figure 2.**
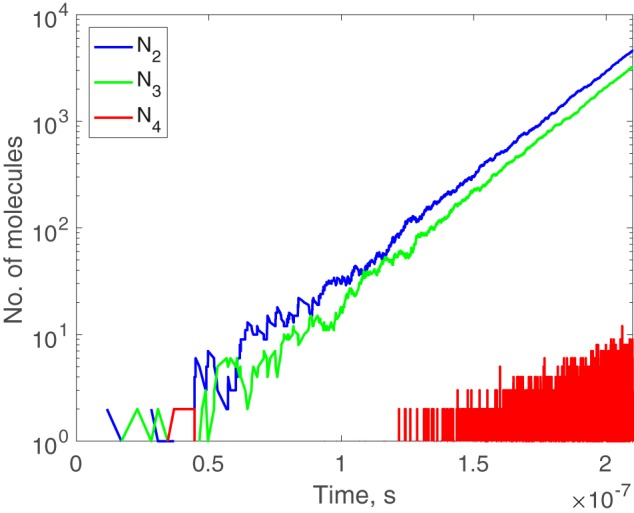
**Dynamics of the formose reaction (Scheme 2) in log-normal scale (*i.e. x* axis is in normal scale and *y* axis is in logarithmic scale).** It is not so clear that *N*_4_(*t*) grows exponentially, because *N*_4_(*t*) is always small. But in solutions of ODEs, we see it clearly (Fig. S3*a*). Note that *N*_4_(*t*) fluctuates frequently between 0 and small numbers, so the curve looks like a block. We set *G*_1_^°^ = 220, *G*_2_^°^ = −760, *G*_3_^°^ −970, *G*_4_^°^ = −1160, *N*_1_(*t*) = *Q*, *N*_2_(0) = 1, and *N*_3_(0) = *N*_4_(0) = 0.

Given the resource molecule 1̄, its dynamics are shown in Fig. 2. All three intermediate molecules (*N*_2_(*t*), *N*_3_(*t*), and *N*_4_(*t*)) increase exponentially. The solutions of the corresponding ODEs are consistent with the simulations (section S6).

The fact that 2̄ grows exponentially can be seen directly by adding up the three low-barrier reactions to obtain 1̄ + 1̄ + 2̄ → 2̄ + 2̄. It indicates that if one 2̄ is present beforehand, one extra 2̄ can be produced, by transforming two of 1̄. Then the additional 1̄ is further used by the system, and more 2̄s are produced. Although the molecules 3̄ and 4̄ are canceled out when we add up the reactions, they also grow exponentially (with 4̄ increasing much more slowly than the other two). The reason is that the actual reaction rate of each low-barrier reaction is not the same (see section S6). This observation is important because it illustrates that it is not just one type of molecule that grows exponentially in such systems, but all of the intermediate molecules.

We define a self-replicating system, of which Scheme 2 is an example, to be a system in which at least one type of molecule is replicated. By investigating various self-replicating systems, we find that not only exponential but also superexponential growth is observed (see examples in section S7). The dynamics indicate that the reactions in self-replicating systems become faster and faster. This is a very special property compared with the collectively catalytic system (*e.g.* the citric acid cycle (Scheme 1)), where the overall reaction rate keeps constant.

In general, the following stoichiometric criteria are sufficient (but not necessary) to show that a physically possible chemical reaction system, given supplies of resource molecules, is self-replicating: 1) the criterion for self-driven (mentioned above) is satisfied; 2) there are some types of intermediate molecules, and the number of times they appear on the reactant side is less than that on the product side (called the *criterion for overproduction*); 3) there is no type of intermediate molecule for which the number of times it appears on the reactant side is larger than that on the product side (called the *criterion for no-overintake*). The formose reaction (Scheme 2) satisfies all of these criteria.

### Effect of side reactions on self-replicating systems

Let us consider the following system (Scheme 3), which is the formose reaction coupled with an additional reaction that transforms 4̄ and 5̄ to 9̄.

$$\begin{array}{c} \{\begin{array}{ccccccc} \bar{1} & + & \bar{2} & \to & \bar{3} & & \\ \bar{1} & + & \bar{3} & \to & \bar{4} & & \\ & & \bar{4} & \to & \bar{2} & + & \bar{2}\\ \bar{4} & + & \bar{5} & \to & \bar{9} & & \end{array}\\ \text{SCHEME}\;3 \end{array}$$

The last reaction can be thought of as a side reaction, which consumes the intermediate molecules.

When there is an infinite reservoir of molecule 1̄, the dynamics of this system are exactly the same as in Fig. 2, because the side reaction 4̄ + 5̄ → 9̄ cannot proceed without 5̄. Even with an infinite reservoir of both molecules 1̄ and 5̄, the dynamics of this system are unaffected, because reaction 4̄ → 2̄ + 2̄ occurs so fast that the side reaction 4̄ + 5̄ → 9̄ cannot obtain molecule 4̄ to proceed. In general, by observing Equations 1 and 3 under “Theory,” decomposition reactions, such as 4̄ → 2̄ + 2̄, occur faster than synthesis reactions, because the reaction rate for synthesis, γ_+_*_ij_*, has an extra term *N_j_*/(*S* + *N*) (compared with γ_−_*_ij_*), which is always smaller than 1. In our experimental setting, the number of solvent molecules *S* is very large, so this extra term is much smaller than 1. In such settings, the self-replicating system outcompetes the side reaction.

Nonetheless, self-replication can be inhibited by some other side reactions. Consider the following system (Scheme 4).

$$\begin{array}{c} \{\begin{array}{ccccccc} \bar{1} & + & \bar{2} & \to & \bar{3} & & \\ \bar{1} & + & \bar{3} & \to & \bar{4} & & \\ & & \bar{4} & \to & \bar{2} & + & \bar{2}\\ \bar{2} & + & \bar{5} & \to & \bar{7} & & \end{array}\\ \text{SCHEME}\;4 \end{array}$$

Given infinite reservoirs of both molecule 1̄ and 5̄, we no longer observe self-replication (*i.e. N*_2_(*t*) and *N*_3_(*t*) do not grow exponentially), because this side reaction consumes the newly produced molecule 2̄ before it can be used by reaction 1̄ + 2̄ → 3̄.

Note that we always assumed ψ*_ij_* = 10 for every low-barrier reaction (see “Theory”), which means that the reaction rate constant βexp(−κΔ*G*_+*ij*_^‡^), or βexp(−κΔ*G*_−*ij*_^‡^), for every low-barrier reaction is identical, denoted as ω. But for now, we relax this assumption. Let the reaction rate constants for the first three reactions in Scheme 4 be identical, ω, and the rate constant for the side reaction be ηω where η ≥ 0. Firstly, when η = 0, the Scheme 4 system goes back to the original formose reaction. Secondly, when the side reaction occurs at low rate (*i.e.* 0 < η < 1), the onset of self-replication is delayed but still occurs eventually (the larger η is, the more delayed the onset of self-replication becomes; figures not shown). Lastly, when the side reaction has a sufficiently high rate (*i.e.* η ≥ 1), it consumes the newly produced molecule 2̄ before it can be used by reaction 1̄ + 2̄ → 3̄, so self-replication is completely inhibited. η = 1 is the critical value in this case.

In the real chemical universe we are living in, the formose reaction is often inhibited by side reactions. Many products further react into a “browning tar” so that the formose reaction has a very low yield of sugars (56). However, this does not preclude the existence of a similar reaction system in the prebiotic world, because, in general, the stability of a self-replicating system depends on the existence and reaction rates of other low-barrier reactions. Whether self-replication is inhibited depends on how side reactions are coupled with the system and what the specific condition is. Also note that all of the arguments above apply to collectively catalytic systems.

### Collectively catalytic and self-replicating systems are common

Another natural question to ask is how common these reaction systems are, such as the citric acid cycle and the formose reaction. Specifically, if we construct alternative chemical universes, or, in another term, different artificial chemistries, where we systematically choose which reactions are low-barrier, we can ask how many of the resulting artificial chemistries contain collectively catalytic or self-replicating systems. To answer this question, we start by using the criterion shared by both of them, that they are self-driven. Table 1 shows the numbers for different *L*, which is set to be the mass of the largest molecule in this particular artificial chemistry in question, to have a measurement of the number of all chemical reaction systems. For example, when *L* = 6, there are in total 1̄, 2̄, …, 6̄, six types of molecules. By choosing which reaction is low-barrier, we can construct ∑_*l* = 0_^9^(_*l*_^9^)·2*^l^* = 19,683 different artificial chemistries, 16,825 of which turn out to be physically possible. Using the criterion for self-driven given above, we find that 6886 (41%) of all of the physically possible artificial chemistries contain self-driven systems. This percentage increases with *L*, which indicates that self-driven systems are common and are more common in systems involving more types of molecules.

**Table 1:** **Number of physically possible artificial chemistries that contain self-driven, collectively catalytic, or self-replicating systems** All of the percentages are calculated with respect to the number of all physically possible artificial chemistries. AC, artificial chemistry; CC, collectively catalytic; SR, self-replicating.

*L*	No. of physically possible ACs*^a^*	No. of ACs containing self-driven systems*^b^*	Lower bound ACs containing CC systems*^c^*	Lower bound ACs containing SR systems*^d^*
4	79	8 (10%)	0 ( 0‰)	2 (25.3‰)
5	681	152 (22%)	5 (7.3‰)	10 (14.7‰)
6	16, 825	6,886 (41%)	21 (1.2‰)	74 (4.4‰)
7	401, 445	232,552 (58%)	184 (0.5‰)	642 (1.6‰)

*^a^* Number of physically possible artificial chemistries.

*^b^* Number of physically possible artificial chemistries that contain self-driven systems.

*^c^* Lower bound on the number of physically possible artificial chemistries that contain collectively catalytic systems.

*^d^* Lower bound on the number of physically possible artificial chemistries that contain self-replicating systems.

However, we cannot be sure that these self-driven systems are collectively catalytic or self-replicating, and there is even a third type of self-driven system, the nonsustaining system, which makes things more complicated (see section S8 for details of the nonsustaining system and section S9 for more details of classification for the self-driven system). Nevertheless, we can use the stoichiometric criteria mentioned above (note that these criteria are sufficient but not necessary) to give a lower bound on the number of artificial chemistries that contain collectively catalytic or self-replicating systems. That is, for a self-driven system, a system is collectively catalytic if it satisfies the criterion for balanced-canceling, or self-replicating if it satisfies both the criteria for overproduction and no-overintake.

Using the stoichiometric criteria, we find that the lower bound on the number of artificial chemistries containing collectively catalytic or self-replicating systems increases with *L*, although the percentage decreases (Table 1). However, because it is the lower bound, it does not mean that the actual number of artificial chemistries containing collectively catalytic or self-replicating systems decreases. Establishing a firm relationship between the number of chemicals (*L*) and self-replication will involve simulating dynamics of all systems.

### How can life maintain low entropy?

According to the second law of thermodynamics, the total entropy of an isolated system never spontaneously decreases over time. Life, thought of as an open system as opposed to an isolated one, is able to maintain order (*i.e.* maintain a relatively low entropy level). Schrödinger suggested that this is achieved by life “feeding on negative entropy” (50). His question is how this can happen spontaneously. But before we answer this, we first need a way to discuss this question concretely and more quantitatively.

Under the framework of our model, if we simply consider life as some self-replicating entity, we should then ask if it is possible for a self-replicating system to spontaneously increase its Gibbs energy or at least keep it unchanged. (We first note that in the scenario of constant pressure and temperature, the decrease of entropy corresponds to the increase of Gibbs energy, because *G* = *H* − *TS*, where *G* is Gibbs energy, *H* is enthalpy, *T* is temperature, and *S* is entropy (51).) Let us consider the self-replicating system in Scheme 5, given the resource molecule 2̄.

$$\begin{array}{c} \{\begin{array}{ccccccc} & & \bar{5} & \to & \bar{1} & + & \bar{4}\\ \bar{2} & + & \bar{3} & \to & \bar{5} & & \\ \bar{2} & + & \bar{4} & \to & \bar{6} & & \\ & & \bar{6} & \to & \bar{3} & + & \bar{3} \end{array}\\ \text{SCHEME}\;5 \end{array}$$

The simulation shows that *N*_1_(*t*), *N*_3_(*t*), and *N*_4_(*t*) increase exponentially, as well as *N*_5_(*t*) and *N*_6_(*t*) (although they are very small; see section S10). Molecule 1̄ is the end product, whereas 3̄, 4̄, 5̄, and 6̄ are replicated through the whole system. We then consider that the “living” system consists of the self-replicating part (namely all of molecules 3̄, 4̄, 5̄, and 6̄) and the resource molecules in the system.

**Figure 3.**
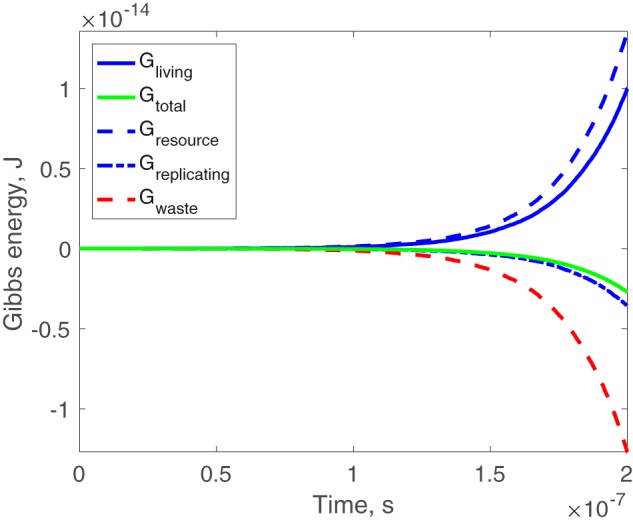
**Time series of Gibbs energy of the self-replicating system of Scheme 5.** We set *G*_1_^°^ = −800, *G*_2_^°^ = −500, *G*_3_^°^ = −400, *G*_4_^°^ = −200, *G*_5_^°^ = −950, *G*_6_^°^ = −750, *N*_2_(*t*) = *Q*, *N*_3_(*0*) = 1, and initially other molecules none.

Now we investigate how Gibbs energy of the system changes. We set Gibbs energy of the initial system to zero (as the reference point), because only relative quantity matters. Therefore, Gibbs energy of the self-replicating part is *G*_replicating_(*t*) = ∑_*i* = 3_^6^*N_i_*(*t*)·*G*_*i*_^°^. Gibbs energy of the resource molecules in the system is *G*_resource_(*t*) = −*F*_2_(*t*)·*G*_2_^°^, where *F*_2_(*t*) is the number of resource molecules 2̄ ever consumed until time *t*. Gibbs energy of the waste is *G*_waste_(*t*) = *N*_1_(*t*)·*G*_1_^°^. Then the Gibbs energy of the living system is *G*_living_(*t*) = *G*_replicating_(*t*) + *G*_resource_(*t*). As shown in Fig. 3, *G*_living_(*t*) increases, whereas *G*_total_(*t*) = *G*_living_(*t*) +*G*_waste_(*t*) decreases.

We have thus given an explicit example of a self-replicating “living” system that spontaneously consumes the resources to increase its own Gibbs energy. Note that our system, as defined, is a well-mixed gas system. So the waste molecules 1̄ are not automatically separated from other molecules, and Gibbs energy of the gas-mixing process is neglected in the calculation above. However, the contribution of the fact of well-mixed gas is relatively small (for details, see section S10). So it is still possible for a self-replicating system to spontaneously increase Gibbs energy or at least keep it unchanged.

### Spontaneous evolution from simple toward complex

Nature provides many examples of the spontaneous evolution from simple toward complex. Is it possible to construct a system showing a similar process? Imagine there is a chemical reaction system composed of the low-barrier reactions listed in Table 2, given an infinite reservoir of only resource molecules 1̄. The first three reactions constitute the formose reaction (Scheme 2), given the resource molecule 1̄. The three reactions in Scheme 8 constitute a collectively catalytic system, given the resource 3̄. The 13 reactions in Scheme 9 constitute a self-replicating system, given the resource 1̄. Is it possible that lots of complex molecules, such 12, 13, and 14, are produced in the end?

**Table 2:**
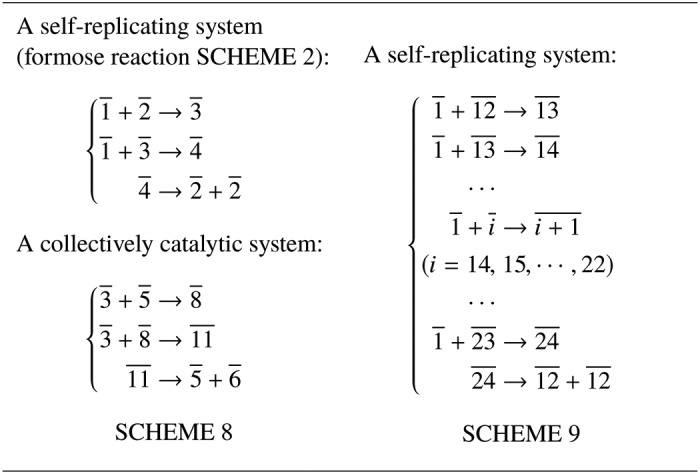
**An example chemical reaction system that is able to evolve from simple toward complex**

The answer in this case is yes. If the first 12 is produced, the self-replicating system of Scheme 9 will be triggered, and consequently *N*_12_ will grow exponentially, as well as *N*_13_, *N*_14_, …, *N*_23_. But how is the first 12 produced? There are actually three stages. 1) Initially, when there are only lots of 1̄s and nothing else, the system stays almost unchanged for a very long time because no low-barrier reaction could occur. Occasionally, by the high-barrier reaction 1̄ + 1̄ → 2̄, one molecule 2̄ is produced. The self-replicating system of Scheme 2 is triggered, and then *N*_2_ and *N*_3_ grow exponentially. Very quickly, there are lots of 2̄ and 3̄. 2) After a relatively long “boring” period, the first 5̄ is produced by the high-barrier reaction 2̄ + 3̄ → 5̄. Then the collectively catalytic system of Scheme 8 is triggered, and *N*_6_ grows. Very soon there are lots of 6̄. 3) After that, occasionally one 12 is produced by the high-barrier reaction 6̄ + 6̄ → 12.

One might naively believe that the first 12 can be produced by other reactions without the need for self-replicating and collectively catalytic systems as Schemes 2 and 8. This is not the case. Despite an abundance of molecules 1̄, which could be used to “assemble” an initial 12, the production of the first 12 requires high-barrier reactions (*e.g.* 6̄ + 6̄ → 12). It is only when there is large number of molecules 6̄ that one such reaction is sufficiently likely to occur. However, without those preceding self-replicating and collectively catalytic systems, molecules 6̄ cannot be sufficiently produced.

The stage-by-stage procedure mentioned above could be generic for how chemical reaction systems evolve toward complex: a relatively simple innovation triggers some self-replicating or collectively catalytic systems and then a large number of new types of molecules are produced, paving the way for other innovations.

Meanwhile, the more types of molecules there are, the more probable it is to have reactions that are high-barrier before becoming practically low-barrier. For example, in the formose reaction (Scheme 2), if the reaction 1̄ + 3̄ → 4̄ is high-barrier, the system is not self-replicating. But imagine that the following three reactions are low-barrier.

$$\begin{array}{c} \{\begin{array}{ccccccc} \bar{1} & + & \bar{30} & \to & \bar{31} & & \\ \bar{3} & + & \bar{31} & \to & \bar{34} & & \\ & & \bar{34} & \to & \bar{4} & + & \bar{30} \end{array}\\ \text{SCHEME}\;6 \end{array}$$

They constitute a collectively catalytic system. Then the reaction 1̄ + 3̄ → 4̄, which is the overall reaction of the three, can still be considered as low-barrier, and the formose reaction system is still self-replicating. The only complication is that we have to wait for the complex molecule 30 to appear.

## Discussion

We have set up a general mathematical model for chemical reaction systems in which molecules are represented by integers, roughly corresponding to the molecules' mass. All chemical reactions are defined by addition operations, which automatically guarantees mass conservation. Each molecule has a predefined standard Gibbs energy of formation, whereas each reaction has a predefined Gibbs energy of activation, which defines the rate constant for each reaction. By changing the Gibbs energy of molecules and reactions, we have investigated a range of artificial chemistries, some of which are like chemical systems found in our real universe. The strength of our approach is that it accounts for the conservation law, energetics, and kinetics while being general enough to investigate the origins of self-replication and the emergence of life.

Although our model did not explicitly include catalysts, as other models did (26, 27, 28), catalysis and autocatalysis emerge in a number of systems. We found three distinct types of self-driven system (*i.e.* systems that “feed” themselves). Both collectively catalytic and self-replicating systems are vital in biology, whereas the third (nonsustaining) system appears less important. In terms of generating complexity, the self-replicating system plays a more important role, because it is able to replicate innovations. For example, in the self-replicating formose reaction (Scheme 2), after the first molecule 2̄ is produced by a high-barrier reaction, more 2̄s are easily replicated. In a biological setting, if this molecule spreads to other places, it can trigger more self-replicating systems. In contrast, in the collectively catalytic citric acid cycle (Scheme 1), for example, after the innovation (the first molecule 5̄), the second 5̄ will not appear until the responsible high-barrier reaction occurs once again.

In biology, most metabolic reactions are catalyzed by sophisticated and highly specific enzymes, and thus avoid disturbance from undesired side reactions. In the very early stage of life, however, probably no reaction required enzymes (11, 15). We found that whether side reactions prevent a system from self-replicating depends on how they are coupled with the system and the specific conditions. There appear to exist examples where self-replication can be sustained in the presence of side reactions. However, how sophisticated enzymes develop *de novo* so that side reactions are avoided is another question, which deserves further investigation.

By arbitrarily constructing different artificial chemistries, we found that lots of them contain self-driven systems, and the lower bounds on the number of artificial chemistries containing collectively catalytic or self-replicating systems increase with more types of molecules. This result suggests that in a random artificial chemistry, it would not be too surprising to observe the emergence of self-replication, one of the central properties of life (1). Although it is not the first theory to propose that self-replication is relatively easy to emerge, as the RAF theory did (29–31), it is the first one requiring no catalyst.

We provided a general model explicitly showing that high thermodynamic free energy molecules can be produced exponentially from low free energy molecules, whereas specific mechanisms in specific real-world scenarios have been investigated before (52, 53). The example system we showed is a metaphor of why high free energy ATP molecules are constantly produced in organisms (14). In addition, as our model takes energetics (corresponding to entropy) into account, it provides a more concrete way to discuss the issue—famously put forward by Schrödinger (50)—why life is able to spontaneously maintain a relatively low entropy level, although it cannot give the full answer. Answering the questions of how the molecules in living systems can be placed in an ordered structure (namely a low entropy state) would require extending our model to include spatial effects.

Our model explicitly shows that complexity evolves from extreme simplicity stage by stage. It gives insights into three issues related to the origin of life. First, the first RNA molecule is much more likely to be produced *de novo* by this stage-by-stage procedure, rather than a magic event (3). It provides theoretical supports to metabolism-first theories (3), such as Wächtershäuser's iron-sulfur world hypothesis (6) and Szathmáry's theory (7–9). Second, before life, Earth should have gone through many stages in which different self-replicating systems existed, and consequently Earth's compositions were different in each stage. The raw materials for life that we should look for are those for the first self-replicating system rather than those for the extant life (6). That is why, in the current theoretical framework, the raw materials for life (*e.g.* nucleotides) seem not to be available on the primordial Earth (3, 4). Third, collectively catalytic and self-replicating systems generate more types of new molecules, and in return, more types of molecules make more reactions feasible (in the form of catalysis and autocatalysis). This could explain why metabolic reactions in extant life always require sophisticated enzymes (15), whereas no reaction is expected to involve catalysts in the very early stage of life (20, 43, 44).

As we noted when introducing the model, in order to be as simple as possible, we currently do not fully account for different isomers or for the impossibility of certain chemical transformations. Nevertheless, the current model can be considered as a simpler version of a more general model, with a more intricate way of “ordering” the complexity of molecules (*e.g.* accounting for different isomers).

Our model provides a convenient platform to construct artificial chemistries and investigate general properties of chemical reaction systems. It may provide a theoretical guideline for systematically searching for other chemical paths toward life (or at least self-replicating entities), as pursued in astrobiology (57, 58) and xenobiology (59) for example.

## Theory

### Constructing chemical reaction systems

We model a well-mixed soup of molecules, each of which is defined by its integer mass, *i*. A molecule's type is thus denoted *ī*. Only two types of reaction are possible: synthesis of two molecules to create a molecule of greater mass (*e.g.* 2̄ + 4̄ → 6̄) and decomposition into two molecules to create two molecules of lower mass (*e.g.* 6̄ → 1̄ + 5̄). All reactions that conserve mass—the total mass on the left-hand side of the equation adds up to those on the right-hand side—can occur. For convenience, we define a reaction pair to be a reaction and its corresponding reverse reaction.

**Figure 4.**
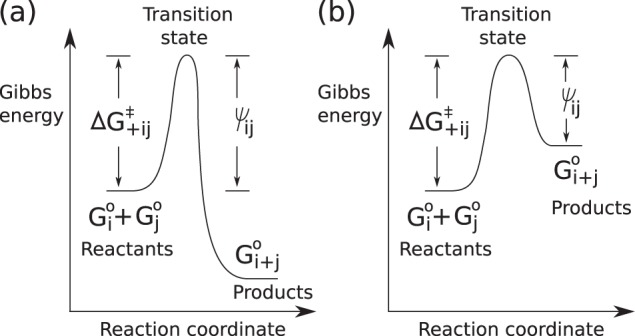
**Diagram of Gibbs energy for a synthesis reaction *ī* + *j̄* → *i* + *j*.** *a*, for the case that the synthesis reaction is spontaneous (*i.e. G*_*i*_^°^ + *G*_*j*_^°^ > *G*_*i* + *j*_^°^). *b*, for the case that it is nonspontaneous (*i.e. G*_*i*_^°^ + *G*_*j*_^°^ ≤ *G*_*i* + *j*_^°^).

Each type of molecule *ī* has its own standard Gibbs energy of formation *G*_*i*_^°^. Then, as illustrated in Fig. 4, a reaction is either spontaneous, meaning that the total standard Gibbs energy of formation of the reactants is greater than that of the products (*i.e. G*_*i*_^°^ + *G*_*j*_^°^ > *G*_*i* + *j*_^°^), or nonspontaneous (*i.e. G*_*i*_^°^ + *G*_*j*_^°^ ≤ *G*_*i* + *j*_^°^). If one reaction in a reaction pair is spontaneous, the other is nonspontaneous, and vice versa.

According to transition state theory (48), the reactants have to overcome the Gibbs energy of activation (namely Δ*G*_+*ij*_^‡^ in Fig. 4) for the reaction to occur. In the model, any reaction pair is either *low-barrier*—the energy barrier (corresponding to ψ*_ij_* in Fig. 4) is low and the reaction rate is thus high—or *high-barrier*. In our system, all reactions are possible, but to define a specific chemical reaction system, we write a list of low-barrier (and spontaneous) reactions only (*e.g.* the citric acid cycle shown in Scheme 1 and the formose reaction shown in Scheme 2.

Although the real citric acid cycle and the formose reaction involve further reactions, in the simplified model above, only carbon-changing reactions are considered. Specifically, in the Scheme 1 system, we consider each reaction uncatalyzed and neglect the fact that each reaction is catalyzed by specific enzymes. Similarly, in the formose reaction, tetroses further aggregate into a “browning tar” whereby the system would be inhibited to some extent (56). We set aside these complexities to have two easy-to-follow special cases, but our framework does, in general, allow us to describe additional side reactions where necessary.

To be as simple as possible, a strong assumption we make is that molecules with the same mass are identical. In real chemistry, molecules with the same mass may have different atomic compositions (*e.g.* CO and N_2_), and even when molecules have the same atomic compositions, they can be different isomers (*e.g.* ethanol and methoxymethane). Furthermore, our model implies that any type of molecule can be produced by some others (because an integer can always be written as a sum of two integers). But in real chemistry, certain chemical transformations are impossible (*e.g.* organometallic compounds can never be produced by a chemical reaction system involved with only carbohydrates). Our model currently excludes these real-world possibilities and limitations. However, we emphasize that the “integer molecule mass” in our model does not necessarily correspond to the physical mass, but should be thought of as a rough ordering of the mass or the relative complexity of molecules.

Note that some chemical reaction systems are not physically possible (*e.g.* the following, shown in Fig. 1*c*).

$$\begin{array}{c} \{\begin{array}{ccccccc} & & \bar{2} & \to & \bar{1} & + & \bar{1}\\ \bar{1} & + & \bar{2} & \to & \bar{3} & & \\ \bar{1} & + & \bar{3} & \to & \bar{4} & & \\ & & \bar{4} & \to & \bar{2} & + & \bar{2} \end{array}\\ \text{SCHEME}\;7 \end{array}$$

By adding up these low-barrier reactions, all molecules are cancelled out. As a consequence, one cannot find proper Gibbs energy for each molecule such that each reaction above is spontaneous. We are only interested in physically possible systems.

### Kinetics

We now specify a general model for the kinetics of our chemical system, under the following assumptions: 1) all molecules are ideally gaseous, 2) the whole system is kept at constant pressure and temperature, and 3) every possible reaction is elementary. The derivation follows the law of mass action and transition state theory (48). Here we cover the key points, and a full derivation is given in section S1.

For any synthesis reaction *ī* + *j̄* → i+j, the reaction rate in unit s^−1^ is as follows,

(Eq. 1) $$\gamma_{+\;ij}=\beta \exp\left(-\kappa \Delta G_{+\;ij}^{‡}\right)\;\cdot \;N_{i}\;\cdot \;N_{j}/\left(S\;+\;N\right)$$

where the subscript +*ij* stands for the synthesis reaction, β and κ are constants, *N_i_* is the number of molecules *ī* in the system, *N_j_* is the number of *j̄*, *S* is the number of solvent molecules, determining the global rate at which molecules interact, *N* is the number of all the molecules except for solvent molecules, and Δ*G*_+*ij*_^‡^, as shown in Fig. 4, is defined as follows.

(Eq. 2) $$\Delta G_{+\;ij}^{‡}=\{\begin{array}{lll} \psi_{ij} & & \text{if}\;G_{i}^{\circ }+G_{j}^{\circ }\;>G_{i\;+j}^{\circ }\\ \psi_{ij}\;+G_{i\;+\;j}^{\circ } & -\left(G_{i}^{\circ }+G_{j}^{\circ }\right) & \text{if}\;G_{i}^{\circ }+G_{j}^{\circ }\;\leq G_{i\;+j}^{\circ } \end{array}$$

When implementing the model, we set values of ψ*_ij_* (positive and finite) for each reaction pair and *G*_*i*_^°^ for each molecule. Together, these give a unique value for Δ*G*_+*ij*_^‡^.

Likewise, for any decomposition reaction *i* + *j* → *ī* + *j̄*, the reaction rate in unit s^−1^ is as follows,

(Eq. 3) $$\gamma_{-\;ij}=\beta \exp\left(-\kappa \Delta G_{-ij}^{‡}\right)\;\cdot \;N_{i\;+\;j}$$

where the subscript −*ij* stands for this decomposition reaction, and the following is true.

(Eq. 4) $$\Delta G_{-\;ij}^{‡}=\{\begin{array}{lll} \psi_{ij} & & \text{if}\;G_{i\;+\;j}^{\circ }>G_{i}^{\circ }\;+G_{j}^{\circ }\\ \psi_{ij}\;+\;\left(G_{i}^{\circ }+G_{j}^{\circ }\right) & -G_{i\;+\;j}^{\circ } & \text{if}\;G_{i\;+\;j}^{\circ }\leq G_{i}^{\circ }\;+G_{i\;+j}^{\circ } \end{array}$$

Because the transition state of a reaction pair *ī* + *j̄* → *i* + *j* and *i* + *j* → *ī* + *j̄* is identical, by setting ψ*_ij_*, both Δ*G*_+*ij*_^‡^ and Δ*G*_−*ij*_^‡^ are uniquely determined. In addition, by setting ψ*_ij_* large or small, we can easily make the reaction pair low-barrier or high-barrier.

### Simulation of dynamics

We take the citric acid cycle (Scheme 1) as an example to illustrate how to set up the simulation experiment. 1) Set up all of the constants. Assume that the constant pressure the system is kept at is 100 kilopascals and the constant temperature is 298.15 K, so we obtain that β ≈ 6.21 × 10^12^ s^−1^ and κ ≈ 0.403 mol/kJ. 2) Set *G*_*i*_^°^ for each type of molecule (up to 6̄ in this case), to make sure that the three reactions are spontaneous (*i.e. G*_5_^°^ > *G*_1_^°^ + *G*_4_^°^, *G*_6_^°^ > *G*_1_^°^ + *G*_5_^°^, and *G*_2_^°^ + *G*_4_^°^ > *G*_6_^°^). Here, we set *G*_1_^°^ = −780, *G*_2_^°^ = −500, *G*_3_^°^ = −490, *G*_4_^°^ = −190, *G*_5_^°^ = −830, and *G*_6_^°^ = −900, all in unit kJ/mol. These values are set in the range of normal chemical substances' Gibbs energy of formation (48, 60), roughly in the range between −1500 and 300. Note that the choice is not unique, and a wide range of choices can be made to allow the system to work. 3) Set ψ_14_ = ψ_15_ = ψ_24_ = 10 for these three low-barrier reactions and ψ*_ij_* = 100 for all other reaction pairs (all in units kJ/mol). 4) Assume that there is an unlimited reservoir of resource molecules. In principle, the resource molecule can be assumed to be any type of molecule, but often to be the types that only appear on the reactant side. In this case, we assume it to be molecule 2̄. In other words, whenever a molecule 2̄ is consumed or produced, it is replenished or removed so that the number of 2̄ always keeps as a constant *Q* = 1000. This setting makes biological sense if we consider a system separated from the unlimited reservoir by a “membrane” or some type of “wall.” As long as some resource molecules are consumed, more will enter the system driven by the chemical gradient. 5) We denote the number of molecule *ī* at time *t* as *N_i_*(*t*). Initially, *N*_4_(0) = 1, and *N*_1_(0) = *N*_3_(0) = *N*_5_(0) = *N*_6_(0) = 0. Molecule 4̄ triggers the chemical reaction system. We also set *S* = 1 × 10^6^, which is much larger than *Q*, so that we are in the dilute limit. We then use the standard Gillespie algorithm to simulate the dynamics (see details in section S2), and the code is accessible (https://github.com/yuernestliu/Self-replication-simulator).^3^ In addition, we construct ODEs to describe the mean-field dynamics (for details, see section S3).

## Author contributions

Y. L. conceptualization; Y. L. software; Y. L. formal analysis; Y. L. and D. J. T. S. supervision; Y. L. funding acquisition; Y. L. validation; Y. L. investigation; Y. L. visualization; Y. L. and D. J. T. S. methodology; Y. L. writing-original draft; Y. L. and D. J. T. S. project administration; Y. L. and D. J. T. S. writing-review and editing.

## Supplementary Material

Supporting Information

## Footnotes

3 Please note that the JBC is not responsible for the long-term archiving and maintenance of this site or any other third party hosted site.

## Footnotes

2 The abbreviations used are:

RAF: reflexively autocatalytic and food-generated
ODE: ordinary differential equation.
