# Supplementary material for: Mathematical modeling reveals spontaneous emergence of self-replication in chemical reaction systems
Source: J Biol Chem. 2018 Oct 3;293(49):18854–63. doi: 10.1074/jbc.RA118.003795 (PMC6295724; doi:10.1074/jbc.RA118.003795)
Supplement: Supporting Information [file supp_293_49_18854__index.html]

Mathematical modeling reveals spontaneous emergence of self-replication in chemical reaction systems — Modeling spontaneous self-replication — Supporting Information 

# Mathematical modeling reveals spontaneous emergence of self-replication in chemical reaction systems

## Supporting Information

- Mathematical and Model Details - SI.pdf
- SI movie for model - SI movie for model
