# Supplementary material for: Mathematical modeling reveals spontaneous emergence of self-replication in chemical reaction systems
Source: J Biol Chem. 2018 Oct 3;293(49):18854–63. doi: 10.1074/jbc.RA118.003795 (PMC6295724; doi:10.1074/jbc.RA118.003795)
Supplement: Supporting Information [file supp_RA118.003795_137897_2_supp_213015_pfzm5m.pdf]

Supporting Information to: Mathematical modeling reveals spontaneous emergence of self-replication in chemical reaction systems

Yu Liu[1] **and David J. T. Sumpter**

Department of Mathematics, Uppsala University, 75105 Uppsala, Sweden

S1. Full derivation of kinetics

We specify a general model for kinetics of our chemical system, under the following assumptions: (1) All molecules are ideally gaseous; (2) the whole system is kept at constant pressure P° and temperature T^*; (3) every possible reaction in the system is elementary.

Reaction rate of synthesis reaction. For synthesis reaction $\bar{i} + \bar{j} \to \overline{i+j}$, its reaction rate is $r_{+ij} = k_{+ij} \cdot [\bar{i}] \cdot [\bar{j}]$, where k_{+ij} is the reaction rate constant, $[\bar{i}]$ is the concentration of component $\bar{i}$, and $[\bar{j}]$ is the concentration of component $\bar{j}$ (1). Note that the reaction rate r_{+ij} is in unit $mol \cdot m^{-3} \cdot s^{-1}$.

We have $[\bar{i}] = n_i/V_{tot}$ where n_i is the amount of $\bar{i}$ and V_{tot} is the volume of the whole system. Since all molecules are ideally gaseous, we apply the ideal gas law $P_{tot} \cdot V_{tot} = n_{tot} \cdot RT$, where P_{tot} is the total pressure of the system, R is the gas constant, T is the temperature of the system, and n_{tot} is the amount of all the molecules in the system. Therefore, $[\bar{i}] = n_i/n_{tot} \cdot P_{tot}/(RT)$. Since the whole system is kept at pressure P° and temperature T^*, that is, $P_{tot} = P^\circ$ and $T = T^*$, we thus have

$$r_{+ij} = k_{+ij}(\frac{P^\circ}{RT^*})^2 \cdot \frac{n_i}{n_{tot}} \cdot \frac{n_j}{n_{tot}}$$

According to transition state theory (1), the reaction rate constant is

$$k_{+ij} = \frac{RT^*}{N_A h}\frac{RT^*}{P^\circ}\exp\left(-\frac{\Delta G^\ddagger_{+ij}}{RT^*}\right)$$

where N_A is the Avogadro constant, h is the Planck constant, and $\Delta G^\ddagger_{+ij}$ is the energy barrier molecule $\bar{i}$ and $\bar{j}$ have to overcome in order for the reaction $\bar{i} + \bar{j} \to \overline{i+j}$ to occur, named as Gibbs energy of activation. Then, we have

$$r_{+ij} = \frac{P^\circ}{N_A h}\exp\left(-\frac{\Delta G^\ddagger_{+ij}}{RT^*}\right) \cdot \frac{n_i}{n_{tot}} \cdot \frac{n_j}{n_{tot}}$$

For simulation, we need the expression of reaction rate in unit s^{-1}, denoted as γ_{+ij}. Therefore, we have

$$\begin{aligned}\gamma_{+ij} &= r_{+ij} \cdot V_{tot} \cdot N_A \\ &= \frac{P^\circ}{N_A h}\exp\left(-\frac{\Delta G^\ddagger_{+ij}}{RT^*}\right) \cdot \frac{n_i}{n_{tot}} \cdot \frac{n_j}{n_{tot}} \cdot \frac{n_{tot}RT^*}{P^\circ} \cdot N_A \\ &= \frac{RT^*}{N_A h}\exp\left(-\frac{\Delta G^\ddagger_{+ij}}{RT^*}\right) \cdot \frac{N_i}{N_A} \cdot \frac{N_j}{N_{tot}} \cdot N_A \\ &= \beta\exp\left(-\kappa\Delta G^\ddagger_{+ij}\right) \cdot N_i \cdot N_j/N_{tot}\end{aligned}$$

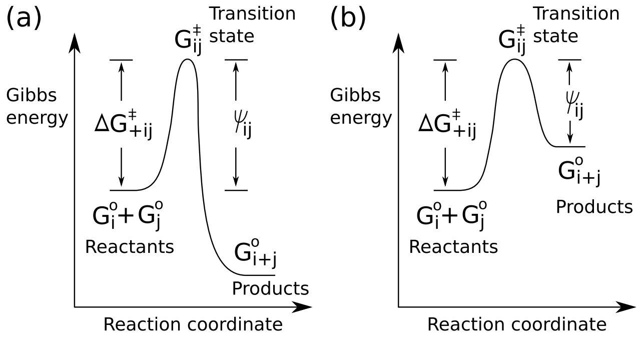

Fig. S1. Diagram of Gibbs energy for a synthesis reaction $\bar{i} + \bar{j} \to \overline{i+j}$. It is similar with Fig. 4 in the main text, but to be clearer, we add $G^\ddagger_{ij}$ in this figure to indicate the Gibbs energy of formation of the transition state. (a) For the case that the synthesis reaction is spontaneous, i.e., $G^\circ_i + G^\circ_j > G^\circ_{i+j}$. (b) For the case that it is non-spontaneous, i.e., $G^\circ_i + G^\circ_j \leqslant G^\circ_{i+j}$.

where $\beta \equiv RT^*/(N_A h)$, $\kappa \equiv 1/(RT^*)$, N_i is the number of molecules $\bar{i}$ in the system, N_j is the number of $\bar{j}$, and N_{tot} is the number of all molecules in the system. Note that the formula is more convenient to use if we separate solvent molecules from other molecules. Here we can consider the solvent molecules as a special type of gaseous molecules $\bar{0}$, which never reacts with any molecule. The number of solvent molecules is denoted by S. Finally, we have

$$\gamma_{+ij} = \beta\exp\left(-\kappa\Delta G^\ddagger_{+ij}\right) \cdot N_i \cdot N_j/(S+N)$$

where $N = \sum_{l>0} N_l$ is the number of all the molecules except for solvent molecules.

Now we explain how to calculate the value of $\Delta G^\ddagger_{+ij}$. Each type of molecule $\bar{i}$ has its own standard Gibbs energy of formation G°_i (the superscript $^\circ$ stands for "standard", i.e., under the pressure P°; the conventional notation of the standard Gibbs energy of formation is $\Delta G^\circ_{f,i}$, but here we denote it simply). So there are two cases, as illustrated in Fig. S1. One is that the total standard Gibbs energy of formation of the reactants is greater than that of the products, namely $G^\circ_i + G^\circ_j > G^\circ_{i+j}$. It is thus a spontaneous reaction. The other case where $G^\circ_i + G^\circ_j \leqslant G^\circ_{i+j}$ is non-spontaneous.

Based on transition state theory (1), $\Delta G^\ddagger_{+ij}$ is determined by a transition state, which can be considered as a short-lived compound associated with molecule $\bar{i}$, $\bar{j}$ and $\overline{i+j}$ (the

[1] To whom correspondence should be addressed. E-mail: yu.ernest.liu@hotmail.com

superscript ‡ denotes a transition state associated). We assume that the transition state also has a specific Gibbs energy of formation $G^{\ddagger}_{ij}$. Then $\Delta G^{\ddagger}_{+ij}$ is defined as the difference of Gibbs energy of formation between the transition state and the reactants, namely $\Delta G^{\ddagger}_{+ij} = G^{\ddagger}_{ij} - (G^{\circ}_i + G^{\circ}_j)$. Instead of using the absolute quantity $G^{\ddagger}_{ij}$ to calculate $\Delta G^{\ddagger}_{+ij}$, we use another relative quantity ψ_{ij}, as shown in Fig. S1. It is defined as the difference between $G^{\ddagger}_{ij}$ and the higher of the two: Gibbs energy of all the reactants and that of all the products. Therefore,

$$\Delta G^{\ddagger}_{+ij} = \begin{cases} \psi_{ij}, & \text{if } G^{\circ}_i + G^{\circ}_j > G^{\circ}_{i+j} \\ \psi_{ij} + G^{\circ}_{i+j} - (G^{\circ}_i + G^{\circ}_j), & \text{if } G^{\circ}_i + G^{\circ}_j \leqslant G^{\circ}_{i+j} \end{cases}$$

Note that ψ_{ij} is always positive and finite. The advantage of using ψ_{ij} instead of $G^{\ddagger}_{ij}$ is that by setting ψ_{ij} large or small, we can easily make the reaction pair $\overline{i}+\overline{j} \to \overline{i+j}$ and $\overline{i+j} \to \overline{i}+\overline{j}$ low-barrier or high-barrier, without considering the relative height of Gibbs energy of formation of the transition state, reactants and products.

Reaction rate of decomposition reaction. The same derivation applies to all the decomposition reactions $\overline{i+j} \to \overline{i} + \overline{j}$. The reaction rate is, in unit $mol \cdot m^{-3} \cdot s^{-1}$,

$$r_{-ij} = k_{-ij} \frac{P^{\circ}}{RT^*} \cdot \frac{n_{i+j}}{n_{tot}}$$

and the reaction rate constant is

$$k_{-ij} = \frac{RT^*}{N_A h} \exp\left(-\frac{\Delta G^{\ddagger}_{-ij}}{RT^*}\right)$$

Note that the unit of k_{-ij} and k_{+ij} are different. That is because $r_{-ij} = k_{-ij}[\overline{i+j}]$ (where $[\overline{i+j}]$ is the concentration of molecules $\overline{i+j}$) while $r_{+ij} = k_{+ij}[\overline{i}][\overline{j}]$, but r_{-ij} and r_{+ij} have the same unit. The ultimate reason is that no matter for a synthesis reaction or a decomposition one, the equilibrium constant is always dimensionless (1).

Likewise, for simulations we need the reaction rate in unit s^{-1}, denoted as γ_{-ij}. Therefore, we have

$$\begin{aligned} \gamma_{-ij} &= r_{-ij} \cdot V_{tot} \cdot N_A \\ &= \frac{P^{\circ}}{N_A h} \exp\left(-\frac{\Delta G^{\ddagger}_{-ij}}{RT^*}\right) \cdot \frac{n_{i+j}}{n_{tot}} \cdot \frac{n_{tot} RT^*}{P^{\circ}} \cdot N_A \\ &= \frac{RT^*}{N_A h} \exp\left(-\frac{\Delta G^{\ddagger}_{-ij}}{RT^*}\right) \cdot \frac{N_{i+j}}{N_A} \cdot N_A \\ &= \beta \exp\left(-\kappa \Delta G^{\ddagger}_{-ij}\right) \cdot N_{i+j} \end{aligned}$$

Note that it has nothing to do with N and S. And we have

$$\Delta G^{\ddagger}_{-ij} = \begin{cases} \psi_{ij}, & \text{if } G^{\circ}_{i+j} > G^{\circ}_i + G^{\circ}_j, \\ \psi_{ij} + (G^{\circ}_i + G^{\circ}_j) - G^{\circ}_{i+j}, & \text{if } G^{\circ}_{i+j} \leqslant G^{\circ}_i + G^{\circ}_j. \end{cases}$$

S2. Gillespie Algorithm

We use the standard Gillespie Algorithm to simulate the dynamics. The steps are as follows:

1. Set up the system as described in the main text section "Theory: Simulation of dynamics". Set $t = 0$.
2. Decide the time interval until the next event, Δt. It is a random number drawn from an exponential distribution with the parameter mean being $1/\gamma_{tot}$ where γ_{tot} is the sum of the reaction rate (in unit s^{-1}) of all the possible reactions in the current step.
3. Randomly choose one reaction. The probability it is chosen is proportional to its reaction rate. Then implement this chosen reaction, i.e., update the number of its reactants and products, and then update reaction rates for all possible reactions.
4. Update time $t = t + \Delta t$, and repeat from step 2.

S3. Ordinary differential equations (ODEs)

We also construct ODEs to describe the mean-field dynamics of a chemical reaction system. Still take the citric acid cycle (Scheme 1 in the main text) as an example, with the same setup. As set, always $N_2(t) = Q$, so we do not need the equation for $N_2(t)$. Then based on the formulas of reaction rates, $N_1(t), N_3(t), N_4(t), N_5(t)$ and $N_6(t)$ can be written as (omit to write the variable t)

$$\begin{cases} dN_1/dt = \omega_{-14} \cdot N_5 + \omega_{-15} \cdot N_6 \\ dN_3/dt = 0 \\ dN_4/dt = -\omega_{+24} \cdot QN_4/(S+N) + \omega_{-14} \cdot N_5 \\ dN_5/dt = -\omega_{-14} \cdot N_5 + \omega_{-15} \cdot N_6 \\ dN_6/dt = -\omega_{-15} \cdot N_6 + \omega_{+24} \cdot QN_4/(S+N) \end{cases} \quad \text{[S1]}$$

where $N = Q + N_1 + N_3 + N_4 + N_5 + N_6$ and $\omega_{\pm ij} = \beta \exp(-\kappa \Delta G^{\ddagger}_{\pm ij})$. For low-barrier reactions, always $\Delta G^{\ddagger}_{\pm ij} = 10\ kJ/mol$, so these $\omega_{\pm ij}$ appeared in Eq. (S1) are all identical, thus denoted as ω. The initial condition for Eq. (S1) is $N_4(0) = 1$ and $N_1(0) = N_3(0) = N_5(0) = N_6(0) = 0$. Then we can numerically solve the equations.

Note that when we construct this equation system, we neglect all the high-barrier reaction pairs since their reaction rates are too small. Also, we neglect the non-spontaneous reaction in each low-barrier reaction pair (e.g., here we did not consider reaction $\overline{6} \to \overline{2} + \overline{4}$), due to the fact that its reaction rate is much smaller than that of the spontaneous one. However, because ψ_{ij} for these reaction pairs is low, if there are lots of reactants present, the reaction rate of the non-spontaneous low-barrier reaction could be comparable with the spontaneous low-barrier one. Therefore, if beforehand we know that there would be lots of reactants of non-spontaneous low-barrier reactions, we have to take them into account when construct the equations.

S4. Detailed dynamics of the citric acid cycle

Fig. S2a shows the dynamics of the citric acid cycle, Scheme 1 in the main text. It shows that the number of molecule $\overline{1}$ increases linearly. The intermediate molecule $\overline{4}, \overline{5}$ and $\overline{6}$ are involved in a cycle of reaction, but the total number of them keeps constant, i.e., always $N_4(t) + N_5(t) + N_6(t) = 1$.

The solutions of the corresponding ODEs are shown in Fig. S2b, which are consistent with the simulation. Note that although $N_1(t)$ shows itself as a straight line, $N_1(t) = at + b$ (where a and b are constants) is actually not an exact solution for the ODEs. The linearity is an approximative and asymptotical behavior.

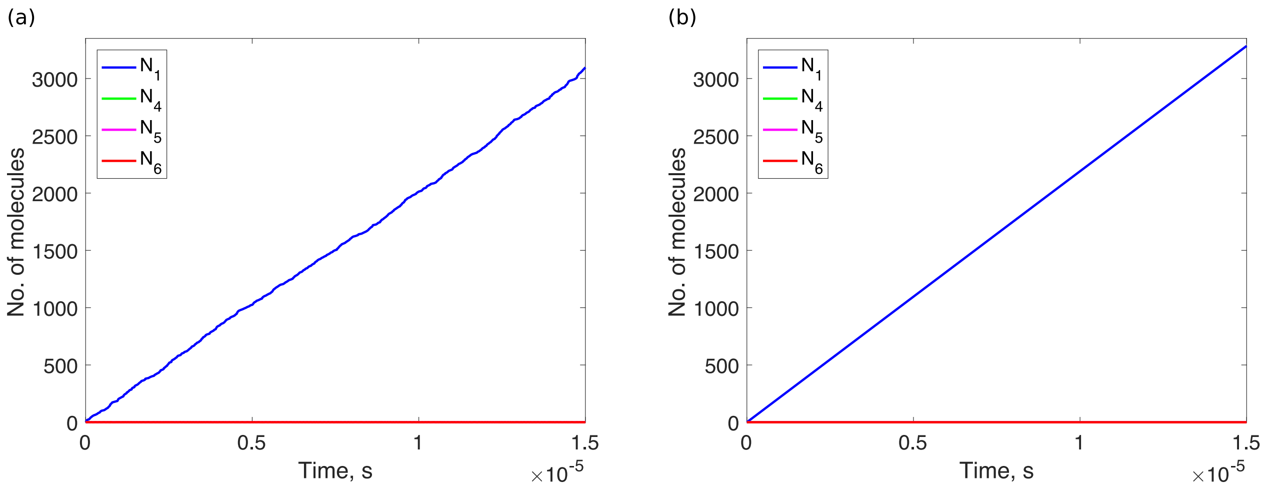

Fig. S2. Dynamics of the citric acid cycle, Scheme 1 in the main text. (a) The outcome of the simulations. The numbers of intermediate molecule $\overline{4}$, $\overline{5}$ and $\overline{6}$ are always 0 or 1, which is hard to see. (b) Solutions of ODEs for this system, with the same setup. After a very short transient time in the beginning, $N_4(t)$, $N_5(t)$ and $N_6(t)$ always stay at around 0.998, 9.96×10^{-4} and 9.96×10^{-4}, respectively, which are very hard to see from the figure.

In the system, reaction $\overline{2} \to \overline{1} + \overline{1}$ itself is high-barrier (namely $\psi_{11} = 100$), so its reaction rate is extremely low (although it is spontaneous since $G_1^\circ = -780$, $G_2^\circ = -500$ and thus $G_2^\circ > G_1^\circ + G_1^\circ$). In this particular experimental setting $\gamma_{-11} \approx 1.88 \times 10^{-2}\ s^{-1}$. However, through these low-barrier reactions, the actual rate of the overall reaction (which can be considered as $\overline{2} \to \overline{1} + \overline{1}$) becomes $1.09 \times 10^8\ s^{-1}$. It is calculated from Fig. S2a, corresponding to half of the slope of the linear curve of $N_1(t)$. It is 5.80×10^9 times larger than the original reaction rate.

S5. Any single catalytic reaction can be written as a collectively-catalytic system

In living systems, many metabolic reactions are catalytic reactions. Although we did not explicitly include catalysts in our model, we find that catalysis is an emergence. For example, the catalytic reaction

$$\overline{2} + \overline{5} \xrightarrow{\overline{4}} \overline{7} \qquad [S2]$$

(meaning that $\overline{4}$ is the catalyst) can be written as the following collectively-catalytic system:

$$\begin{cases} \overline{2} + \overline{4} \to \overline{6} \\ \overline{11} \to \overline{4} + \overline{7} \\ \overline{5} + \overline{6} \to \overline{11} \end{cases} \qquad [S3]$$

In fact, any single catalytic reaction can be written as a collectively-catalytic system. In order to show this, we need to discuss the three general types of reactions: synthesis, decomposition and replacement reactions.

Firstly, a catalyzed synthesis reaction can be generally written as

$$A + B \xrightarrow{C} (AB)$$

where A stands for a type of molecule (the same for other capital letters), and (AB) stands for a compound consisting of A and B. If we write this reaction step by step, it can be written as Eq. (S4), which is actually a collectively-catalytic system. We can always work out a one-to-one representation of the molecules. One example (often the representation is not unique) is Eq. (S5), where $\overline{2}$ stands for A, $\overline{3}$ for B, $\overline{4}$ for C, $\overline{5}$ for (AB), $\overline{6}$ for (AC) and $\overline{9}$ for (ABC), respectively.

$$\begin{cases} A + C \to (AC) \\ B + (AC) \to (ABC) \\ (ABC) \to C + (AB) \end{cases} \qquad [S4] \qquad\qquad \begin{cases} \overline{2} + \overline{4} \to \overline{6} \\ \overline{3} + \overline{6} \to \overline{9} \\ \overline{9} \to \overline{4} + \overline{5} \end{cases} \qquad [S5]$$

Secondly, a catalyzed decomposition reaction can be generally written as

$$(DE) \xrightarrow{F} D + E$$

Then it can be written as a collectively-catalytic system Eq. (S6), and one example of the one-to-one representation of the molecules is shown in Eq. (S7), where $\overline{2}$ stands for F, $\overline{4}$ for E, $\overline{5}$ for D, $\overline{7}$ for (DF), $\overline{9}$ for (DE) and $\overline{11}$ for (DEF), respectively.

$$\begin{cases} (DE) + F \to (DEF) \\ (DEF) \to (DF) + E \\ (DF) \to D + F \end{cases} \qquad [S6] \qquad\qquad \begin{cases} \overline{9} + \overline{2} \to \overline{11} \\ \overline{11} \to \overline{7} + \overline{4} \\ \overline{7} \to \overline{5} + \overline{2} \end{cases} \qquad [S7]$$

Lastly, a catalyzed replacement reaction can be generally written as

$$G + H \xrightarrow{P} J + K$$

Then it can be written as a collectively-catalytic system Eq. (S8). Note that there are conservations between the reactants and products, e.g., the mass sum of G and H is equal to the mass sum of J and K. That is why we have the reaction $(GPH) \to (PJ) + K$ in Eq. (S8). One example of the one-to-one representation of the molecules is shown in Eq. (S9), where $\overline{3}$ stands for J, $\overline{4}$ for G, $\overline{5}$ for H, $\overline{6}$ for K, $\overline{7}$ for P, $\overline{10}$ for (PJ), $\overline{11}$ for (GP) and $\overline{16}$ for (GPH), respectively.

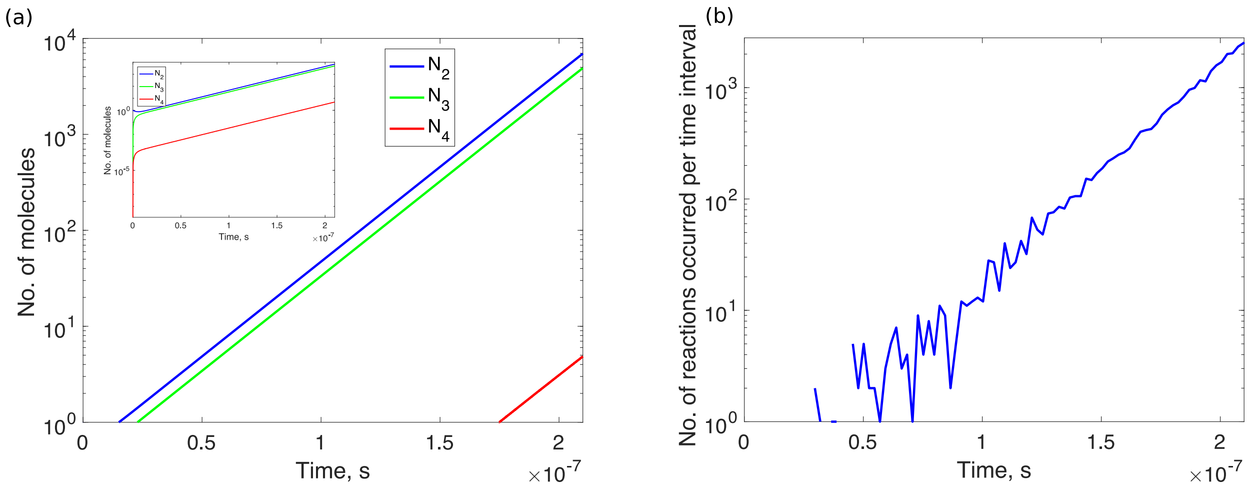

Fig. S3. Dynamics of the formose reaction, Scheme 2 in the main text. (a) Solutions of ODEs in log-normal scale, i.e., x-axis is in normal scale and y-axis is in logarithmic scale. It only shows the solutions larger than 10^0, where we are interested as the number of molecules can only be natural numbers. The inset shows the complete solutions. (b) The number of reactions occurred in the system per time interval 2.28×10^{-9} s (this value is chosen such that there are 100 data points in this figure). In the beginning, the reaction occurs slowly. So for some time intervals in the beginning, there is no reaction occurred, and consequently, the corresponding y-coordinates should be $\log_{10}(0) = -\infty$. Those corresponding points are not drawn. That is why it is sometimes discontinuous in the beginning.

$$\begin{cases} G + P \to (GP) \\ (GP) + H \to (GPH) \\ (GPH) \to (PJ) + K \\ (PJ) \to P + J \end{cases} \quad \text{[S8]}$$

$$\begin{cases} \overline{4} + \overline{7} \to \overline{11} \\ \overline{11} + \overline{5} \to \overline{16} \\ \overline{16} \to \overline{10} + \overline{6} \\ \overline{10} \to \overline{7} + \overline{3} \end{cases} \quad \text{[S9]}$$

Note that for metabolic reactions in living systems, the catalysts such as enzymes are always highly specific, i.e., one type of enzyme always only catalyzes one specific reaction. For example, if the reaction Eq. (S2) is a metabolic reaction, the catalyst $\overline{4}$ most likely only interacts with $\overline{2}$ even though there are lots of other types of molecules around. Therefore, if we want to replace Eq. (S2) with the collectively-catalytic system Eq. (S3), we should make sure that $\overline{4}$, $\overline{6}$ and $\overline{11}$ do not interact with other molecules in this chemical universe.

S6. Detailed dynamics of the formose reaction

For the formose reaction (Scheme 2 in the main text), we can also construct ODEs. As set, always $N_1(t) = Q$. Then,

$$\begin{cases} dN_2/dt = \omega(-QN_2/(S+N) + 2N_4) \\ dN_3/dt = \omega(-QN_3/(S+N) + QN_2/(S+N)) \\ dN_4/dt = \omega(-N_4 + QN_3/(S+N)) \end{cases} \quad \text{[S10]}$$

The solutions are shown in Fig. S3a, which are consistent with the simulation. We also observe that $N_4(t)$ grows exponentially but is kept at small numbers (two or three magnitudes fewer than $N_2(t)$ and $N_3(t)$). That is why it is hard to see the exponential growth in Fig. 2 in the main text. Note that the exponential growth of $\overline{2}$ (namely $N_2(t) = c \cdot e^{\alpha t}$), as well as that of $\overline{3}$ and $\overline{4}$, is not an exact solution for Eq. (S10) (we can clearly see the super-exponential growth at the beginning, from the inset of Fig. S3a). The exponential dynamics is an approximative and asymptotical behavior.

The overall reaction of the system is helpful to understand the system. In this case the number of times each low-barrier reaction occurs is not the same, so the overall reaction can only be obtained by adding up these reactions weighted by the number of times of occurrence. Till the end of the simulation, reaction $\overline{1} + \overline{2} \to \overline{3}$ occurs 24253 times, reaction $\overline{1} + \overline{3} \to \overline{4}$ occurs 17133 times, and reaction $\overline{4} \to \overline{2} + \overline{2}$ occurs 17126 times. Then we have

$$\begin{aligned} &(24253 + 17133) \cdot \overline{1} + 24253 \cdot \overline{2} + 17133 \cdot \overline{3} + 17126 \cdot \overline{4} \\ &\to (17126 \times 2) \cdot \overline{2} + 24253 \cdot \overline{3} + 17133 \cdot \overline{4} \end{aligned}$$

where the numbers before each molecule represent how many times it appears. Then we cancel $24253 \cdot \overline{2}$, $17133 \cdot \overline{3}$ and $17126 \cdot \overline{4}$ on both sides, and get the overall reaction

$$41386 \cdot \overline{1} \to 9999 \cdot \overline{2} + 7120 \cdot \overline{3} + 7 \cdot \overline{4}$$

We find that there are net productions of molecule $\overline{2}$, $\overline{3}$ and $\overline{4}$. That is why $N_2(t)$, $N_3(t)$ and $N_4(t)$ grow exponentially, rather than $N_2(t)$ alone.

Fig. S3b shows the number of reactions occurred during every certain time interval. It explicitly shows that the reactions become faster and faster.

S7. Dynamics of a super-exponential growing self-replicating system

Exponential growth is one of the dynamics self-replicating systems have. Some other self-replicating systems have super-exponential growth. Take the following self-replicating system (given the resource molecule $\overline{6}$) as an example,

$$\begin{cases} \overline{1} + \overline{1} \to \overline{2} \\ \overline{3} \to \overline{1} + \overline{2} \\ \overline{5} \to \overline{1} + \overline{4} \\ \overline{2} + \overline{6} \to \overline{8} \\ \overline{8} \to \overline{3} + \overline{5} \end{cases} \quad \text{[S11]}$$

Similarly, we setup the system: $G_1^\circ = -150$, $G_2^\circ = -530$, $G_3^\circ = -410$, $G_4^\circ = -1170$, $G_5^\circ = -570$, $G_6^\circ = -230$, $G_7^\circ = -950$,

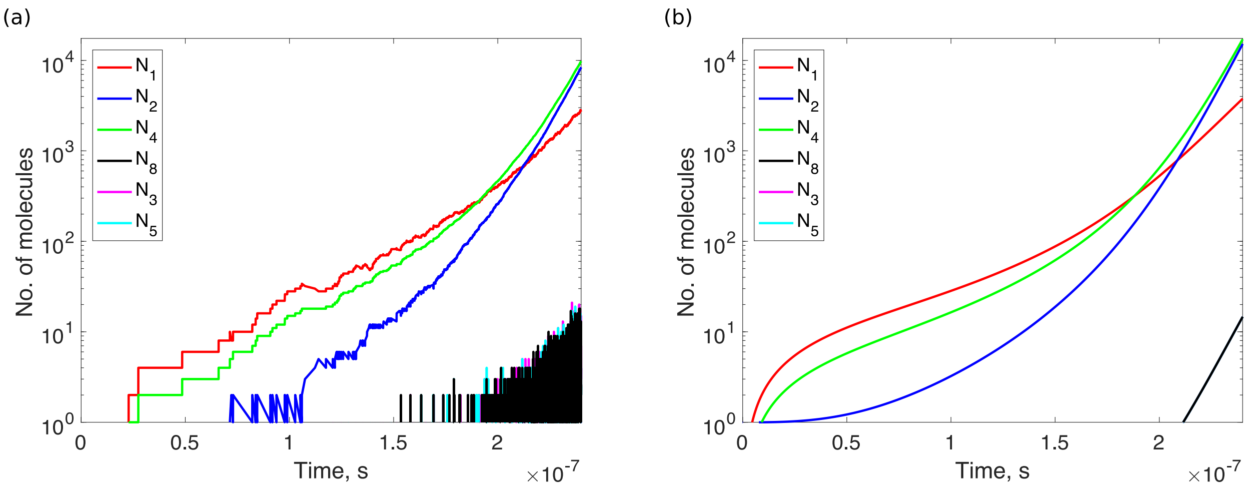

Fig. S4. Dynamics of the self-replicating system Eq. (S11) in log-normal scale. (a) Results of simulations. (b) Solutions of the ODEs. It only shows the solutions larger than 10^0, where we are interested. Note that $N_8(t)$, $N_3(t)$ and $N_5(t)$ are always the same.

$G_8^\circ = -780$, $N_6(t) = Q$, $N_2(0) = 1$ and $N_1(0) = N_3(0) = N_4(0) = N_5(0) = N_7(0) = N_8(0) = 0$.

The results of simulation are shown in Fig. S4a. We see that $N_1(t)$, $N_2(t)$ and $N_4(t)$ grows faster than exponential. As shown in Fig. S4b, the solutions of the corresponding ODEs are consistent with the simulations.

S8. Non-sustaining system

Here we show an example of the non-sustaining system (another group of self-driven system). The system is Eq. (S12), given the resource molecule $\overline{4}$,

$$\begin{cases} \overline{1}+\overline{1} \to \overline{2} \\ \overline{1}+\overline{3} \to \overline{4} \\ \quad\ \overline{6} \to \overline{1}+\overline{5} \\ \overline{2}+\overline{4} \to \overline{6} \\ \quad\ \overline{6} \to \overline{3}+\overline{3} \end{cases} \qquad [S12]$$

Similarly, we setup the system: $G_1^\circ = -130$, $G_2^\circ = -370$, $G_3^\circ = -770$, $G_4^\circ = -1050$, $G_5^\circ = -1490$, $G_6^\circ = -1430$, $N_4(t) = Q$, $N_2(0) = 1$ and $N_1(0) = N_3(0) = N_5(0) = N_6(0) = 0$.

The dynamics (figure not shown) shows that these low-barrier reactions cannot practically occur. So the system cannot proceed in the time scale of occurrence of low-barrier reactions. The reason is that: even if $\overline{4}$ is supplied, the system still needs $\overline{2}$ to proceed; while the molecules $\overline{2}$ produced by reaction $\overline{1}+\overline{1} \to \overline{2}$ are not enough to fully provide the molecule $\overline{2}$ that is needed. Even if we set $N_2(0) = 100$, the system will still become practically static after $\overline{2}$ is consumed up. We call this kind of self-driven system as non-sustaining system.

S9. Stoichiometry and classification for self-driven systems

Stoichiometry. In general, stoichiometry is not enough to discern whether a self-driven system is collectively-catalytic, self-replicating, or non-sustaining (instead, one has to check its dynamics). There are two examples of such systems.

The first one is the chemical reaction system Eq. (S13), given the resource molecule $\overline{3}$. By checking the stoichiometry,

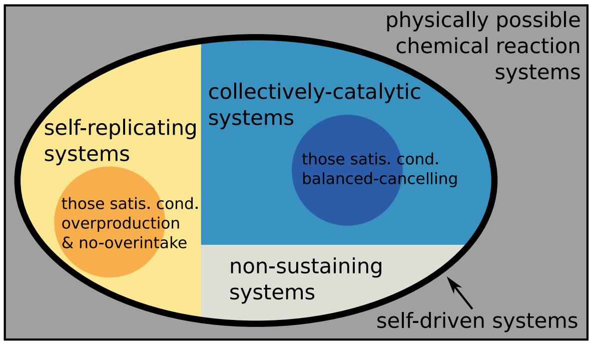

Fig. S5. The classification for self-driven systems. The dark blue disk represents the collectively-catalytic systems which satisfy the criterion for balanced-cancelling. The dark yellow disk represents the self-replicating systems which satisfy the criteria for overproduction and for no-overintake.

we find that it satisfies the criterion of self-driven. But it does not satisfy the criterion for balanced-cancelling, since the number of times the intermediate molecule $\overline{1}$ appears on the reactant side and that on the product side are different, as well as for $\overline{4}$. So we cannot discern whether it is collectively-catalytic. Now we check the criteria for overproduction and no-overintake. The number of times the intermediate molecule $\overline{1}$ appears on the reactant side is less than that on the product side, which means that the criterion for overproduction is satisfied; while the number of times the intermediate molecule $\overline{4}$ appears on the reactant side is larger than that on the product side, which means that the criterion for no-overintake is violated. Therefore, we cannot discern whether this system is self-replicating neither.

After checking its dynamics, we find that it is collectively-catalytic. The mechanism underlying is that the number of times the low-barrier reaction $\overline{1}+\overline{3} \to \overline{4}$ occurred is twice as many as that of the other three low-barrier reactions (those three reactions occur for the same number of times). So in order to obtain the overall reaction, we should weight $\overline{1}+\overline{3} \to \overline{4}$ twice, as shown in Eq. (S14). The overall reaction is then obtained by adding up these reactions, that is, $\overline{3}+\overline{3} \to \overline{6}$

Table S1. Number of physically possible artificial chemistries that contain self-driven, collectively-catalytic or self-replicating systems

L	Cho.[1]	0	1	2	3	4	5	6	7	8	9	10	11	12	Total
4	All[2]	1	8	24	32	16	-	-	-	-	-	-	-	-	81
	Phy[3]	1	8	24	32	14	-	-	-	-	-	-	-	-	79
	SD[4]	0	0	0	4	4	-	-	-	-	-	-	-	-	8
	CC[5]	0	0	0	0	0	-	-	-	-	-	-	-	-	0
	SR[6]	0	0	0	2	0	-	-	-	-	-	-	-	-	2
5	All	1	12	60	160	240	192	64	-	-	-	-	-	-	729
	Phy	1	12	60	160	234	168	46	-	-	-	-	-	-	681
	SD	0	0	0	12	52	64	24	-	-	-	-	-	-	152
	CC	0	0	0	4	1	0	0	-	-	-	-	-	-	5
	SR	0	0	0	4	5	1	0	-	-	-	-	-	-	10
6	All	1	18	144	672	2,016	4,032	5,376	4,608	2,304	512	-	-	-	19,683
	Phy	1	18	144	672	2,002	3,892	4,822	3,584	1,446	244	-	-	-	16,825
	SD	0	0	0	28	290	1,144	2,168	2,082	988	186	-	-	-	6,886
	CC	0	0	0	10	6	5	0	0	0	0	-	-	-	21
	SR	0	0	0	9	27	29	9	0	0	0	-	-	-	74
7	All	1	24	264	1,760	7,920	25,344	59,136	101,376	126,720	112,640	67,584	24,576	4,096	531,441
	Phy	1	24	264	1,760	7,894	24,928	56,208	89,806	99,550	74,328	35,602	9,874	1,206	401,445
	SD	0	0	0	52	860	5,866	21,596	47,266	64,132	54,652	28,614	8,438	1,076	232,552
	CC	0	0	0	28	57	79	17	3	0	0	0	0	0	184
	SR	0	0	0	12	85	190	189	103	53	10	0	0	0	642

[1] Number of low-barrier reactions chosen.
[2] Number of all alternative chemical universes, in another term, different artificial chemistries.
[3] Number of physically possible artificial chemistries.
[4] Number of physically possible artificial chemistries that contain self-driven systems.
[5] Lower bound on the number of physically possible artificial chemistries that contain collectively-catalytic systems.
[6] Lower bound on the number of physically possible artificial chemistries that contain self-replicating systems.

$$\begin{cases} \overline{2} \to \overline{1} + \overline{1} \\ \overline{1} + \overline{3} \to \overline{4} \\ \overline{8} \to \overline{2} + \overline{6} \\ \overline{4} + \overline{4} \to \overline{8} \end{cases} \quad \text{[S13]}$$

$$\begin{cases} \overline{2} \to \overline{1} + \overline{1} \\ \overline{1} + \overline{3} \to \overline{4} \\ \overline{1} + \overline{3} \to \overline{4} \\ \overline{8} \to \overline{2} + \overline{6} \\ \overline{4} + \overline{4} \to \overline{8} \end{cases} \quad \text{[S14]}$$

collectively catalyzed by the intermediate molecule $\overline{1}$, $\overline{2}$, $\overline{4}$ and $\overline{8}$.

The second example is the following self-driven system, given the resource molecule $\overline{2}$,

$$\begin{cases} \overline{1} + \overline{1} \to \overline{2} \\ \overline{4} \to \overline{1} + \overline{3} \\ \overline{2} + \overline{3} \to \overline{5} \\ \overline{2} + \overline{5} \to \overline{7} \\ \overline{7} \to \overline{3} + \overline{4} \end{cases}$$

For the intermediate molecule $\overline{1}$, the number of times it appears on the reactant side is larger than that on the product side, so the criteria for balanced-cancelling and for no-overintake are violated. We thus cannot discern just by stoichiometry whether this system is collectively-catalytic or self-replicating. By checking its dynamics, it turns out to be a self-replicating system.

Classification. For a chemical reaction system, we can discern whether it is self-driven just by stoichiometry, as the definition. But as we said, generally we need to check its dynamics to discern whether it is collectively-catalytic, self-replicating, or non-sustaining. Fortunately, a self-driven must be collectively-catalytic if it satisfies the criterion for balanced-cancelling; while it must be self-replicating if it satisfies the criteria for overproduction and for no-overintake. Fig. S5 is a diagram for the complete classification.

Now we investigate how common these systems are. As we have mentioned in the main text, we define L to be the mass of the largest molecule in the particular artificial chemistry in question. Then there are L types of molecules $\overline{1}$, $\overline{2}$, ..., $\overline{L}$, and $L^2/4$ reaction pairs if L is even or $(L^2-1)/4$ reaction pairs if L is odd. For example $L=6$, then there are 9 reaction pairs. Note that (1) we are interested in the artificial chemistries that contain self-driven, collectively-catalytic or self-replicating systems, rather than the artificial chemistries that themselves are self-driven or etc., and (2) although by stoichiometry, the exact numbers of such systems cannot be obtained, we can at least give a lower bound on those numbers. Table S1 shows the numbers for different L (a detailed version of Table 1 in the main text).

S10. Dynamics of the self-replicating system where entropy is decreased, Scheme 5 in the main text

For the self-replicating system Scheme 5 in main text, results of simulation and solutions of the corresponding ODEs are shown in Fig. S6. They are consistent with each other. We see that $N_5(t)$ and $N_6(t)$ also increase exponentially, but they are always extremely small (in this case, always under 10).

Now we show how Gibbs energy of the system changes with taking into account the Gibbs energy contribution of the gas-mixing process. Here we first presume that molecule $\overline{1}$ (the waste) is separated from other molecules. In the constant temperature and pressure scenario, the Gibbs energy of mixing perfect gases is given by the formula (1)

$$G_{mix} = \frac{RT}{N_A} D \sum_i \left(\frac{D_i}{D} \ln \frac{D_i}{D}\right)$$

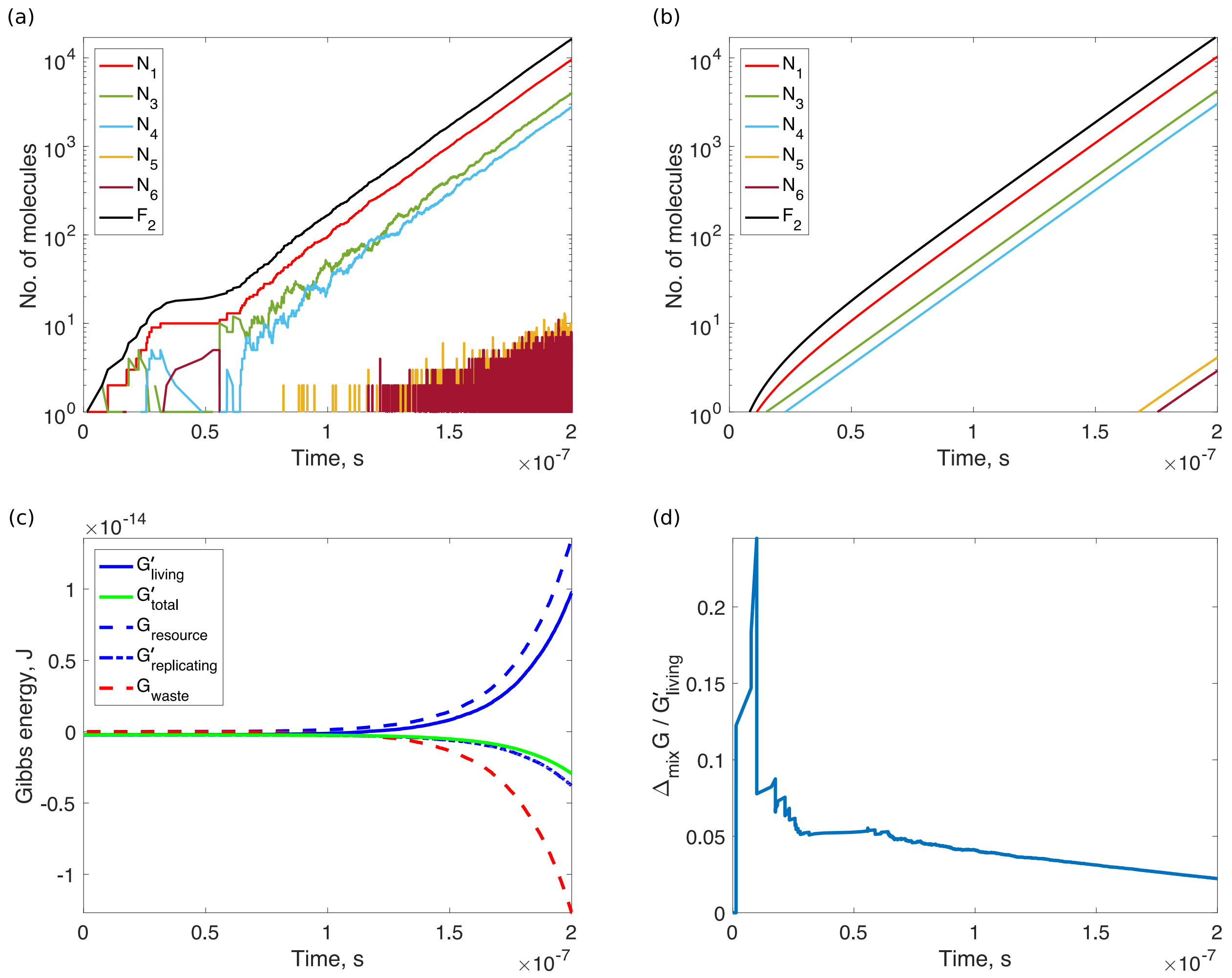

Fig. S6. Dynamics and Gibbs energy of the self-replicating system Scheme 5 in the main text. (a) Results of simulations. (b) Solutions of the ODEs. It only shows the solutions larger than 10^0, where we are interested. (c) Time series of Gibbs energy of the system, with taking into account the Gibbs energy contribution of the gas-mixing process. This figure is very similar with Fig. 3 in the main text. (d) Time series of $\Delta_{mix}G(t)/G'_{living}(t)$.

where D_i represents the number of gas component i and $D = \sum_i D_i$ is the sum. So in this system, Gibbs energy of the self-replicating part gets additional terms

$$G'_{replicating}(t) = \sum_{i=3}^{6} N_i(t) \cdot G_i^\circ + \frac{RT^*}{N_A} B(t)\left(\frac{S}{B(t)} \ln \frac{S}{B(t)} + \sum_{i=2}^{6} \frac{N_i(t)}{B(t)} \ln \frac{N_i(t)}{B(t)}\right)$$

where $B(t) = S + \sum_{i=2}^{6} N_i(t)$, and S, as we introduced before, is the number of the solvent molecules (in this case, $S = 1 \times 10^6$). The other Gibbs energy $G_{resource}(t)$ and $G_{waste}(t)$ stay the same as in the main text, $G'_{living}(t) = G'_{replicating}(t) + G_{resource}(t)$, and $G'_{total}(t) = G'_{living}(t) + G_{waste}(t)$. Fig. S6c shows the time series of Gibbs energy. They have very similar trends as Fig. 3 in the main text.

Now we go back to the actual situation where molecules $\bar{1}$ are not automatically separated from other molecules. In this case, energy is needed to separate them. The minimum energy needed is the Gibbs energy difference before and after mixing molecules $\bar{1}$ with others (denoted as $\Delta_{mix}G$), and we have

$$\Delta_{mix}G(t) = \frac{RT^*}{N_A} B(t)\left(\frac{S}{B(t)} \ln \frac{S}{B(t)} + \sum_{i=2}^{6} \frac{N_i(t)}{B(t)} \ln \frac{N_i(t)}{B(t)}\right) - \frac{RT^*}{N_A} C(t)\left(\frac{S}{C(t)} \ln \frac{S}{C(t)} + \sum_{i=1}^{6} \frac{N_i(t)}{C(t)} \ln \frac{N_i(t)}{C(t)}\right)$$

where $C(t) = S + \sum_{i=1}^{6} N_i(t)$. Fig. S6d shows the times series of $\Delta_{mix}G(t)/G'_{living}(t)$. We see that it is always very small, i.e., always $G'_{living}(t) > \Delta_{mix}G(t)$. So, it is possible that some portion of the living system's Gibbs energy is used to separate molecule $\bar{1}$ (although here we do not talk about how). Therefore, we can still conclude that it is possible for a self-replicating system to spontaneously increase its Gibbs energy or at least keep it unchanged.

1. Atkins P, de Paula J (2014) *Atkins' physical chemistry.* (Oxford University Press), 10th edition, pp. 185, 894–900.
